# Cardiac Autonomic Nervous System Activity during Slow Breathing in Supine Position

**DOI:** 10.1155/2021/6619571

**Published:** 2021-02-27

**Authors:** Satoru Kai, Koji Nagino, Takuma Aoki, Tatsuya Imura, Keita Kiyoshima, Yoshinobu Satake, Kaname Matsuura, Kota Mima, Shoki Yasuoka, Akinobu Yabuuchi

**Affiliations:** Division of Physical Therapy, Department of Rehabilitation Sciences, Faculty of Allied Health Sciences, Kansai University of Welfare Sciences, 3-11-1 Asahigaoka, Kashiwara City, Osaka 582-0026, Japan

## Abstract

The purpose of this study is to clarify cardiac autonomic nervous system activity during slow breathing exercises in a supine position. Eighteen healthy young males were participated. Heart rate variability was measured for 5 minutes at rest, 5 minutes at slow breathing, and then 5 minutes at rest. As a result, the LF/HF ratio increased with slow breathing, but HF value did not change. We suggest that the increased LF/HF ratio may be due to increased airway resistance. Cardiac autonomic nervous system activity during slow breathing in the supine position was revealed.

## 1. Introduction

Heart rate variability (HRV) is a popular research tool for assessing cardiac autonomic nervous system (ANS). HRV has also been used in studies to monitor the recovery of heart rate after exercise [1] and to reveal changes due to different postures [2–7]. However, HRV has not been used in studies that reveal cardiac ANS activity during exercise intervention in a supine position. Therefore, it is unclear how exercise intervention in the supine position affects cardiac ANS activity.

With aging, overall cardiac ANS activity and vagal nerve activity decrease. Therefore, it is desired to develop a method for activating cardiac ANS activity. Previous studies have reported that cardiac ANS activity is enhanced by interval training and slow breathing exercise [1, 8]. However, disuse disorders occur due to aging; it is necessary to ensure safety and security by appropriate methods. Since physiotherapists often examine patients in the supine position, we will clarify the response of cardiac ANS activity to the subjects' supine position due to slow breathing load. The important thing is to clarify cardiac ANS activity during exercise intervention in the supine position and apply it to the physiotherapy program. Therefore, first, the purpose of this study is to clarify cardiac ANS activity during slow breathing in the supine position.

## 2. Materials and Methods

Eighteen healthy young males (age 20 ± 1 years (mean ± S.D.), height 171 ± 5 cm, body mass 68 ± 18 kg, and BMI 23 ± 6 kg·m^−2^) were included. To clarify the effect of slow breathing in the supine position, rest was performed in the supine position for 5 minutes, slow breathing in the supine position for 5 minutes, and then resting in the supine position for 5 minutes. Slow breathing was performed under the conditions of exhalation at 6 seconds and inspiration at 4 seconds. Diet, smoking, caffeine intake, and alcohol consumption that affect autonomic nervous activity were prohibited from 2 hours before the measurement. The measurement was carried out in an environment where the room temperature was 24.0 to 26.6°C and the humidity was 50.8 to 61.8%. To investigate cardiac ANS activity, an electrocardiogram electrode was attached to the chest, and the data were wirelessly sent to a PC for analysis. The electrocardiogram was monitored by binary light recorder (GMS Company) during the experiment for HRV analysis. Power spectra obtained from spectral analysis were defined as two components: 0.04∼0.15 Hz (low frequency (LF)) and 0.15∼0.4 Hz (high frequency (HF)). HF power almost entirely mediated by vagal nerve activity [9] and was affected by the vagal nerve activity generated by respiration [10, 11]. LF power reflects the mixed modulation of vagal and sympathetic nerve activities [12]. The ratio of LF power to HF power (LF/HF) was considered to reflect the sympathovagal balance, and high values suggested a sympathetic predominance [13]. Therefore, according to previous studies, HF power was used as an index of vagal nerve activity, and LF/HF ratio was used as an index of sympathovagal balance. HF power and LF/HF ratio were logarithmically transformed and shown as ln HF and ln LF/HF. LF power and LF + HF values, which indicate other autonomic nervous activities, were also used as indicators, and the logarithmically transformed ln LF and ln LF + HF values were also used as indicators.

For statistical analysis, SPSS for windows ver. 22.0 (SPSS Inc.) was used, and after the normality test, one-way analysis of variance with repeated measures and multiple comparison test were performed. The significance level was 5%.

Informed consent was obtained from all individual participants included in the study. All procedures performed in this study were in accordance with the ethical standards of the Kansai University of Welfare Sciences Human Research Ethics Committee and with the 1964 Helsinki Declaration and its later amendments or comparable ethical standards.

## 3. Results

The results of each value at rest, slow breathing, and subsequent rest in the supine position are presented in Table 1.

The heart rate values at rest, slow breathing, and subsequent rest in the supine position were 75 ± 10 at rest, 76 ± 9 at slow breathing, and 73 ± 8 at subsequent rest. There was no statistically significant difference among the three groups.

## 4. Discussion

Slow breathing in the supine position revealed that sympathovagal balance activity was activated and vagal nerve activity was unchanged. In the previous study [2], the value of the ln LF/HF ratio at rest in the supine position was 0.2 ± 0.8 for males and −0.3 ± 0.9 for females. In the results of this study, it is 0.8 ± 0.9, which is a little higher, but it can be seen that it is at the same level. In addition, the ln HF value was 3.8 to 6.2 in the previous study [2–7], and 5.5 ± 1.2, which is the result of this study, is within that range. The values during slow breathing compared with the values at rest increased to 2.5 ± 0.5 in the ln LF/HF ratio, and a statistically significant difference was observed. Ln HF value decreased to 5.0 ± 1.1, and a statistically significant difference was observed. Slow breathing increased LF, ln LF, LF + HF, and ln LF + HF values, all of which were statistically significant. These values indicate an increase in cardiac ANS activity other than the HF value because the HF value has neither increased nor decreased. These findings indicate that slow breathing in the supine position stimulates sympathovagal balance activity.

It has been found that slow breathing excites vagal nerve activity and reduces the heart rate, with vagal nerve activity predominant [14–16]. Slow breathing has a remarkable impact on cardiac control above and beyond vagal modulation [17]. In addition, the vagal nerve activity is further enhanced by lengthening the exhalation time rather than the inspiration time during slow breathing [15, 16]. Decreasing the breathing rate increases the magnitude of heart rate variability and respiratory sinus arrhythmia [18]. Increases regularity of heart rate variability suggesting a decreased complexity of cardiac control [19]. Slow breathing suppresses adrenal medulla norepinephrine secretion and sympathetic nerve excitement. Nevertheless, slow breathing in the supine position does not stimulate vagal nerve activity, but stimulates sympathovagal balance activity. However, the load is low, and in the previous study, the ln LF/HF ratio in the resting position was around 2 [1], and the ln LF value in the resting supine position was in the range of 3.7 to 7.2 [2–7]. The sitting ln LF value is in the range 5.2-7.7 [5, 20, 21]. In the results of this study, the ln LF/HF ratio and ln LF value during slow breathing are 2.5 and 7.5, which are close to the values compared with sitting at rest in previous studies. From these results, it is considered that the load on sympathovagal balance is not high. Therefore, slow breathing in the supine position is likely to be a therapeutic exercise that can also be used for elderly people.

Controlled breathing maneuver is used to assess cardiovascular autonomic control [22–27]. Previous studies have verified that decreased breathing rate is responsible for the increased cardiac vagal regulation [28–33]. Results associating breathing control maneuvers with lower breathing rates and the improvement of vagal modulation are still contradictory [33]. A controlled breathing protocol is used as a method of inducing vagal nerve activation. However, an increase in cardiac sympathetic component in healthy subjects at slow breathing (0.1 Hz) and an improvement in vagal component at faster breathing pattern (0.2 Hz) have been observed [18, 34]. The results of these previous studies support the results of this study.

As an immediate response, cardiac ANS activity is unlikely to occur even if slow breathing is performed in a stressed state in a sitting position [8]. Continued slow breathing training improves autonomic function [14–16]. The results of those previous studies are divided. Slow breathing increased vagal nerve activity and decreased sympathovagal balance activity [15, 16], but no change in sympathetic nerve activity [14]. Resting blood pressure changes with slow breathing for 4 weeks [36]. Slow breathing in 55 healthy subjects reduces both systolic and diastolic blood pressures [37].

When the body position changes, various physiological changes occur in the body. When changing from an upright position to a recumbent position, total lung capacity, vital capacity, and residual capacity are generally slightly reduced, but the expiratory reserve is significantly reduced and a compensatory increase in inspiratory reserve is inadequate [38]. Pulmonary mixing is inferior in the supine position than in the upright position [39, 40]. Mechanical resistance during inspiration is lower at slow respiratory rates and higher in the supine position than in the sitting position [41]. We suggest that mechanical resistance during expiration is higher at slow respiratory rates than at fast respiratory rates, and at slow respiratory rates in the supine position is higher than in the sitting position. In slow respiratory rates in the supine position, during expiration has a higher mechanical resistance than during inspiration. The effects of such airway resistance may also have stimulated the sympathetic nerve activity. The lung is difficult to expand in the supine position. That may also have affected airway resistance. Details will be clarified in the future.

## 5. Conclusions

Slow breathing in the supine position of young healthy males was found to stimulate cardiac ANS activity without HF power. Especially that, the LF/HF ratio increased. We suggest that the increased LF/HF ratio may be due to increased airway resistance. In the future, we will measure in elderly people with or without disease to clarify the effect of cardiac ANS activity during slow breathing in the supine position.

## Figures and Tables

**Table 1:** Resting, slow breathing, and subsequent resting values in the supine position.

	Rest	Slow breathing	Subsequent rest
HF	449 ± 381	449 ± 381	305 ± 260
ln HF	5.5 ± 1.2^∗^	5.0 ± 1.1	5.3 ± 0.9
LF/HF ratio	4.3 ± 3.5^∗^	15.7 ± 7.8	4.5 ± 2.7^∗^
ln LF/HF ratio	0.8 ± 0.9^∗^	2.5 ± 0.5	1.0 ± 0.7^∗^
LF	926 ± 818^∗^	2607 ± 1891	1110 ± 1222^∗^
ln LF	6.3 ± 0.9^∗^	7.5 ± 0.9	6.3 ± 1.1^∗^
LF + HF	1375 ± 1066^∗^	2877 ± 2108	1415 ± 1462^∗^
ln LF + HF	6.8 ± 0.9^∗^	7.5 ± 0.9	6.7 ± 0.9^∗^

^∗^Slow breathing vs. *p* < 0.05, multiple comparison test.

## Data Availability

There is no data availability.
